# Catechol-O-methyltransferase activity in individuals with substance use disorders: a case control study

**DOI:** 10.1186/s12888-022-04068-x

**Published:** 2022-06-21

**Authors:** Jacinto Nuno da Costa Azevedo, Cláudia Carvalho, Maria Paula Serrão, Rui Coelho, Margarida Figueiredo-Braga, Maria Augusta Vieira-Coelho

**Affiliations:** 1 Department of Neuroscience and Mental Health, Faculty of Medicine, University of Porto, Rua da Alegria 2083, 4200-027 Porto, Portugal; 2 i3S – Institute for Research and Innovation in Health, University of Porto, Rua da Alegria 2083, 4200-027 Porto, Portugal; 3 Department of Biomedicine-Therapeutics and Pharmacology Unit, Faculty of Medicine, University of Porto, Porto, Portugal; 4 Centro Hospitalar Universitário São João, Porto, Portugal

**Keywords:** Impulsivity, Substance use disorders (SUD), S-COMT activity, Case-control study

## Abstract

**Background:**

Impulsivity and substance use disorders (SUD) have been both associated with changes in dopaminergic processes. In this study, we intended to evaluate the dopaminergic function in imprisoned SUD offenders through the determination of s-COMT activity.

**Methods:**

The study included 46 male individuals from a Portuguese penal institution. The participants were assessed through a battery of standardised instruments: Psychopathy Checklist-Revised (PCL-R), Barratt Impulsivity Scale Version 11 (BIS-11), and the European version of the Addiction Severity Index (EuropASI). In addition, s-COMT erythrocyte activity was evaluated.

**Results:**

Overall, 73.9% (*n* = 34) of the individuals had Antisocial personality disorder (ASPD) and 58.7% (*n* = 27) presented SUD. We evidenced, for the first time, that, in individuals with SUD, s-COMT activity was correlated with the severity of drug dependence (EuropASI) (*p* = 0.009), and with BIS-11 factors self-control (*p* < 0.0001) and non-planning (*p* = 0.002).

**Conclusions:**

This study opens new perspectives regarding the pharmacological intervention on substance dependence through the interference on dopamine pathways.

## Introduction

Impulsivity has been recognized as a multi-dimensional construct associated with an array of psychiatric disorders [1]. It results in a tendency to engage in inappropriate, maladaptive or poorly conceived behaviors characterized by absence of forethought or consideration of outcomes, delayed gratification, novelty-seeking, impatience, short attention span, and difficulty to persiste at a particular activity [2]. Behavioral, neurobiological, and imaging techniques have evidenced that impulsive behaviors are associated with alcohol and substance abuse, both as a vulnerability markers and as a consequences [2, 3]. In fact, substance users are known to be highly impulsive and present a variety of well identified symptoms: impaired control, social impairment, risky use, and pharmacological tolerance and withdrawal [2].

Even though clinical evidence shows that impulsivity and substance use disorders (SUD) can coexist and can be related to the same environmental factors [3], this complex relationship shall be analyzed considering three main factors: i) impulsivity as a trait, with focus on decreased cognitive and response inhibition; ii) the effect of acute or chronic substance use on brain structure and function; and iii) genetic and environmental factors [3, 4]. In this context, from a neurobiological perspective, both impulsivity and substance abuse have been associated with changes in dopaminergic processes suggesting that these conditions may be regulated not only by environmental factors but also by biological ones. In individuals with SUD, it has been evidenced that increased brain dopamine concentrations, in limbic brain regions, are responsible for reinforcing effects [5]. However, the mechanisms involved in SUD are far more complex and rely also on structural changes of the prefrontal cortex (PFC) with reduction of the activity of this region, through a mechanism of downregulation of dopamine receptors type 2 (DRD2), in the striatum, resulting in impaired self-control and impulsive behaviours [6]. On the other hand, research indicates that impulsivity is also modulated by the levels of dopamine in the PFC and may be understood as an imbalance of bottom-up and top-down neural systems suppressed by automatic or reward-driven responses with diminished cognitive control to demands [3, 7]. All these mechanisms are part or result from the brain reward cascade, in which dopamine plays a central role [8]. The reward cascade involves the release of serotonin, which in turn stimulates enkephalin, at the hypothalamus; enkephalin inhibits GABA at the substantia nigra, which tunes the amount of dopamine released at the nucleus accumbens, also known as “reward site” [8]. When dopamine is released into the synapse, it stimulates dopamine receptors (D1-D5) resulting in increased feelings of well-being and stress reduction. As such, dopamine has been called the “pleasure molecule” and/or the “antistress molecule.” In this scenario, it is not surprising that dysfunctions in the brain reward cascade, which can be caused by genetic variants mainly in the dopaminergic system, cause a reduction in dopamine levels with the development of multiple drug-seeking behaviors aiming to increase dopamine levels to achieve feelings of well-being [8].

One of the most immediate ways to assess these mechanisms is focusing on dopamine metabolism in the PFC and limbic brain, with particular emphasis on the enzyme catechol-O-methyltransferase (COMT) [9]. In fact, COMT is the primary mechanism for dopaminergic inactivation in the PFC [10] and is widely distributed in human brain. It is directly involved in the regulation of synaptic dopamine concentrations in prefrontal neurons [10]. In the striatum, this mechanism is regulated through neuronal uptake of dopamine by abundant specific transporters. In this context, low COMT activity would result in increased concentration of dopamine at the synapse and, therefore, in higher action at the dopamine receptors of the receiving neuron. However, several studies evidenced an inverted-U-shaped relationship between extracellular dopamine concentration and global PFC network activity [11, 12], so that both excessive and insufficient levels may impair cognitive performance. This could suggest the existence of a COMT mediated mechanism through which optimal levels of dopamine would modulate signal gain to support cognitive functioning.

Soluble COMT (s-COMT) activity has already been assessed in the setting of impulsivity and SUD with the objective of clarifying the physiological mechanisms underlying these disorders [3, 13]. We postulate that s-COMT activity may be related to high impulsivity and dependence severity due to impaired self-control.

Considering that addictive behaviors may also have a genetic component, genes related with dopamine synthesis, degradation, receptors, and transporters are possible research topics, as a complement to COMT activity assessment [14]. In this context, the COMT Val^158^Met (rs4680) polymorphism has already been studied in the scope of substance addiction with evidence of a significant correlation with SUD [15, 16]. The COMT Val^158^Met polymorphism determines the dopamine concentration in the prefrontal cortex (PFC), but not in the striatum [17]. In fact, we can find only a few DRD2 receptors in the PFC [18]. However, they are abundant in the striatum, meaning that, to evaluate the dopaminergic component of addiction, dopamine receptors shall also be investigated. In this setting, several studies have shown that Taq I A1 allele of the DRD2 gene is associated with alcoholism, substance abuse, and other impulsive behaviors and personality traits, so that it became a topic of research during the characterization of individuals with SUD [14, 19].

Even though the dopaminergic pathways of SUD have already been studied, as far as we know, this kind of evaluation has never been performed along with a full psychiatric evaluation, including anxiety, depression, antisocial personality disorder (ASPD), suicidal attempts, impulsivity, psychopathy measurement, and evaluation of addiction related variables. This kind of extensive evaluation is essential to fully characterize the population, identify unknown correlations, and validate SUD diagnosis since it enables to distinguish between SUD and other disorders with similar manifestations, like ASPD. In incarcerated populations, substance abuse and mental disorders, like ASPD, are expressive and, as such, prisons are an adequate environment to collect data that can clarify the neurobiology of this association and improve the search for novel therapeutic approaches [20].

In this study, we intended to further clarify the biological mechanisms underpinning SUD and impulsivity, in incarcerated individuals, through the evaluation of s-COMT activity along with an extensive psychiatric evaluation.

## Materials and methods

### Study population

This study was conducted in a medium/high security penal institution for male offenders, in the North of Portugal. At the time of the protocol application, the penitentiary institution had a total number of 710 inmates, which had received sentences longer than 10 years.

Our sample included 46 male inmates, recruited by a convenience sampling strategy, between January and March 2015. Participants were referred to the Psychiatry clinical services due to SUD and as a part of a methadone treatment plan. Participants should also be over 18 years old. The ability to read and provide written informed consent was also taken into consideration. Participation was voluntary and no reward was offered in exchange for participation. The participants were able to leave the research at any time without any consequences; the individuals who decided not to participate received the same treatment offered to participants. In accordance with the Declaration of Helsinki, written, informed consent was obtained after explaining the procedures to each participant. The study followed a case-control design.

The research protocol was formally approved by an Ethics Committee from Centro Hospitalar e Universitário de São João (Document number 48.14) and by the hosting institution, the General Directorate for Probation and Prison Services.

### Procedures and instruments

The participants that agreed to participate were interviewed and went through a blood sample collection, in the clinical department of the penitentiary institution.

Diagnosis was performed by a forensic psychiatrist (JA) using a standardised interview – the Mini-International Neuropsychiatric Interview (MINI) [21], which provided information on SUD, anxiety, and depression. In this study, we resorted to the Portuguese version of MINI [22]. The psychometric assessment of participants included a battery of standardised instruments: the Psychopathy Checklist-Revised (PCL-R) [23], the Barratt Impulsivity Scale Version 11 (BIS-11) [24], and the European version of the Addiction Severity Index (EuropASI) [25]. The only biological variable was S-COMT erythrocyte activity.

Participants and scorers were blinded to the results of the s-COMT assay.

### Psychopathy checklist-revised (PCL-R)

Psychopathy was assessed through the PCL-R, which measures psychopathic traits by collecting information from clinical records and applying a semi-structured interview. The 20 items that compose PCL-R are scored as absent (0), present to some degree (1), or fully present (2), providing a maximum total score of 40 points. The PCL-R is a four-factor model comprising interpersonal, affective, lifestyle, and antisocial facets. The reliability of PCL-R was recently re-evaluated (Cronbach’s alpha value: total score = 0.87, factor 1 = 0.86, facet 1 = 0.77, facet 2 = 0.79, factor 2 = 0.86, facet 3 = 0.79, facet 4 = 0.79) [26]. The structural properties of PCL-R were previously validated in Portuguese samples with a kappa index of 0.87 and a sensitivity measure of 84.8% [27, 28].

### Barratt impulsiveness scale version 11 (BIS-11)

The BIS-11 is a self-report questionnaire used for assessing general impulsivity [24]. The current scale version contains 30 items, which are rated from 1 (rarely/never) to 4 (almost always/always). Factor analyses reveal six first-order factors (attention, cognitive instability, motor, perseverance, self-control–reverse scoring–, and cognitive complexity), and three second-order factors (attentional, motor, and non-planning). The structural properties of BIS-11 were replicated in Portuguese-speaking subjects [29]. In this study, we used the Portuguese version of BIS-11, which was recently re-evaluated with the following Cronbach’s alpha values for each score: BIS-11 total score = 0.84, attention = 0.80, cognitive instability = 0.62, motor = 0.84, perseverance dimension = 0.53, self-control = 0.80, and cognitive complexity = 0.67 [30].

### European version of the addiction severity index (EuropASI)

The EuropASI was applied to access the severity of SUD. This semi-structured interview offers an inventory of problems that occurred over the previous month in six areas: physical health, work income, substance use, legal status, family and social relationships, and psycho-emotional status. The EuropASI also assesses history of suicide attempts and criminality type. This multidimensional clinical and research instrument is an adapted version of the Addiction Severity Index (5th version) [25].

The composite scores of each dimension ranged from 0 to 1, while higher scores indicated greater severity. The reliability measures indicated moderate to good internal consistency in the European samples (Cronbach’s alpha: 0.69–0.92) [31].

### Erythrocyte soluble catechol-O-methyltransferase (s-COMT) assay

Erythrocyte s-COMT was obtained from washed erythrocytes submitted to haemolysis as described elsewhere [32]. Venous blood samples were collected between 08.00 and 09.00 a.m., after an overnight fast, and kept on ice in K_3_EDTA tubes, until processing. Blood samples were centrifuged at 1500 g for 10 minutes at 4 °C, the plasma was removed; the uppermost cell layer was separated for genetic analysis. Afterwards, a volume of cold 0.9% NaCl solution was added to the erythrocytes and gently vortexed. Thereafter, the tubes were centrifuged (at 1500 g, 10 minutes, 4 °C) and the supernatant discarded. This process was repeated twice. Washed erythrocytes were stored at − 70 °C, until the enzyme assay was carried out. On the day of the experiment, the frozen erythrocytes were thawed on ice.

Haemolysis was conducted at a ratio of 4:1 (water:erythrocytes; *V*:*V*). Following vigorous mixing, the tubes stood on ice for 10 minutes. Then, the tubes were centrifuged at 20,000 g, for 20 minutes at 4 °C, and the supernatant was collected for the assay of erythrocyte s-COMT. The protein content was determined using human serum albumin as standard [33].

s-COMT activity was determined by the ability of enzyme preparations to methylate adrenaline to metanephrine. The reaction was stopped with perchloric acid. The samples were kept at 4 °C for 2 hours, and then centrifuged (5400 g, 10 minutes, 4 °C); 500 mL aliquots of the supernatant, filtered on 0.22 mm pore size Spin-X filter tubes (Costar), were used for the assay of metanephrine.

### Assay of catechol derivatives

Metanephrine was determined by HPLC with electrochemical detection, in aliquots of samples from the COMT assay, as previously described by Vieira-Coelho [34]. In each assay, aliquots of 20 or 50 mL were injected into the chromatographic system by means of an automatic sample injector (Gilson 231) connected to a Gilson dilutor (Gilson 401). The chromatographic system included a pump (Gilson 307) and a stainless steel 5 mm ODS2 column (Biophase; Bioanalytical Systems, West Lafayette, IN, USA) of 25 cm length and 4.6 mm diameter. The degassed mobile phase was pumped at a rate of 1.0 mL/min; it was composed of citric acid 0.1 mM, sodium octylsulphate 0.5 mM, sodium acetate 0.1 M, Na2EDTA 0.17 mM, dibutylamine 1 mM, and methanol (10% v/v); pH was adjusted to 3.5 with PCA 2 M. Detection was performed electrochemically by means of a glassy carbon electrode, an Ag/AgCl reference electrode, and an amperometric detector (Gilson 142); the detector operated at 0.75 V. Gilson Unipoint HPLC software was used to monitor the produced current. The detection limit of metanephrine ranged from 350 to 1000 fmol.

### Drugs

S-adenosyl-L-methionine, DL-metanephrine and adrenaline (bitartrate salt) were purchased from Sigma Chemical Co. (St Louis, MO).

### DNA extraction

Genomic DNA extraction from leukocytes was performed following the manufacturer’s instructions of the Quick-DNA Plus Kits (Zymoresearch, CA, USA). A 100 ng/μL DNA aliquot was stored at − 80 °C until use.

### Genotyping

Allelic discrimination for the COMT Val^158^Met (rs4680) and DRD2/ANKK1 TaqIA (rs1800497) polymorphisms were determined through real-time polymerase chain reaction (PCR) technique, using a TaqMan SNP genotyping assay with fluorogenic probes (Applied Biosystems, Foster City, CA). Briefly, 15 ng of DNA was amplified in a total volume of 8 μL containing 0.2 μL of a minor groove binder (MGB) probe solution (Applied Biosystems) and 4 μL of TaqMan universal polymerase chain reaction master mix (Applied Biosystems). PCR conditions were provided by the manufacturer: 40 cycles of 95 °C denaturation (15 sec), 60 °C anneal/extension (1 min).

Thermal cycling and fluorescence signal genotyping were performed through the StepOnePlus Real-Time PCR system (Applied Biosystems, Foster City, CA). A positive control for each possible genotype and a negative control were included in each 96-well plate.

### Statistical analysis

Data were summarised using descriptive statistics: continuous variables are presented as mean ± standard deviation (s-COMT activity) or median (interquartile range [Q1, Q3]; age, education level, length of imprisonment, BIS-11, PCL-R, EuropASI, days of heroin and cannabis use, and methadone dosage), while categorical variables (frequency of violent crimes, ASPD, depressive disorders, psychopathy, anxiety disorders, suicide attempts, SUD, COMT Val158Met (met/met and met/val), and A1 allele of DRD2 receptors) are presented as absolute or relative frequencies. Continuous variables were subjected to normality testing (Kolmogorov–Smirnov test). In data with normal distribution t-Student Test was used to compare means. Multiple comparisons were performed via Bonferroni test. When data did not meet the requirements of parametric tests, non-parametric Mann–Whitney was used. A *p* value < 0.05 was considered to be statistically significant.

The degree of association between variables without normal distribution was measured by the Spearman correlation coefficient.

Analyses were carried out using IBM SPSS Statistics for Mac, Version 26.0 (Armonk, NY, USA: IBM Corp.).

## Results

### Study population

Our sample comprised 46 individuals with a median age of 36.0 (33.0, 42.0). The median education level was 6.0 (6.0, 9.0), while the median length of imprisonment, at the time of the assessment, was 102.0 (60.0, 166.0) months. A total of 54.3% (*n* = 25) of the inmates had been convicted for violent crimes (physical assault, murder, or attempted murder). Overall, 73.9% (*n* = 34) of the individuals had ASPD, 58.7% (*n* = 27) presented SUD, 21.7% (*n* = 10) had depressive disorders, 30.4% (*n* = 14) had anxiety disorders, 43.5% (*n* = 20) exhibited psychopathy, and 32.7% (*n* = 15) had personal histories of suicide attempts.

The sample was divided in two groups according to the presence or absence of SUD, based on the diagnosis performed with the MINI interview, at the beginning of the study (Table 1). The two groups were comparable on age, education, time spent in prison, frequency of violent crimes, presence of depressive and anxiety disorders, and history of suicide attempts (Table 1). Individuals with SUD had a higher frequency of ASPD and a higher score in the PCL-R F3-lifestyle parameter (*p* = 0.020), than individuals without SUD (Table 1). The presence of COMT polymorphisms (63.2% vs 66.7%, *p* = 0.330) and the frequency of A1 allele of DRD2 receptors (21% vs 33%, *p* = 0.430) were similar in both groups.

**Table 1:** Sociodemographic and criminal characteristics, and psychiatric assessment of participants with and without SUD

	No SUD (***n*** = 19)	SUD (***n*** = 27)	*p*-value
Age (years)	35.0 (28.0, 39.0)	35.0 (31.0, 43.0)	0.070
Education (years)	6.0 (6.0, 10.0)	8.0 (6.0, 9.0)	0.446
Length of imprisonment (months)	120.0 (60.0, 174.0)	96.0 (60.0, 180.0)	0.797
Violent crimes	12 (63.2%)	13 (48.1%)	0.310
ASPD	11 (57.9%)	23 (85.2%)	**0.038**
Depressive disorders	6 (31.6%)	14 (51.8%)	0.170
Anxiety disorders	5 (26.3%)	9 (33.35)	0.610
Suicide attempts	4 (21.1%)	11 (40.7%)	0.160
Psychopathy	6 (31.6%)	4 (14.8%)	0.180
PCL-R total	25.0 (18.0, 32.0)	28.0 (25.0, 33.0)	0.290
F1-Interpersonal	4.0 (4.0, 8.0)	8.0 (4.0, 8.0)	0.130
F2-affective	5.0 (2.0, 8.0)	6.0 (2.0, 8.0)	0.950
F3-Lifestyle	8.0 (5.0, 10.0)	10.0 (8.0, 10.0)	**0.020**
F4-Antisocial	6.0 (5.0, 10.0)	8.0 (4.0, 10.0)	0.700

Results are presented as median (Q1, Q3) or mean ± SD for continuous variables and n (%) for categorical variables

*ASPD* Antisocial Personality Disorder; *PCL-R* Psychopathy Checklist-Revised, *SUD* Substance Use Disorder

Individuals with SUD were divided in two groups, one including participants with active substance consumption (56%) and other in which participants were not consuming any kind of substance (44%). Table 2 provides a comparison of the clinical characteristics of participants with SUD (with and without active consumption) and without SUD. The median age of first consumption in the overall SUD group was 14 (10, 16) years and was similar in both groups (active consumption vs no active consumption) (Table 2). The BIS-11 first order factor self-control was significantly different between the three groups (*p* = 0.034). The participants that were still in active substance consumption reported to have consumed cannabis and heroin, in 20 and 27 days of the previous month, respectively. In this group, SUD were mostly associated with heroin and the individuals were reported to the psychiatry consultation as part of the protocol of methadone treatment. s-COMT activity was similar in both groups (with and without active consumption) and was not statistically different from that of non-SUD individuals (Table 2). All the parameters regarding SUD characterization (EuropASI factors and methadone dosage) were also similar in both groups (Table 2).

**Table 2:** Comparison of the clinical characteristics of participants with SUD (with and without active consumption) and without SUD

	Without SUD (***n*** = 19)	SUD No consumption (***n*** = 15)	SUD Consumption (***n*** = 12)	***p*** value
**Age at first consumption (years)**	–	15 (12, 17)	10 (9, 16)	0.093
**BIS-11 Total**	53.0 (43.0, 70.0)	58.0 (46.0, 64.0)	65.0 (56.5, 70.5)	0.082
*First order factors*				
Attentional	9.0 (5.0, 11.0)	9.0 (6.0, 11.0)	9.0 (8.0, 10.5)	0.370
Cognitive instability	5.0 (3.0, 7.0)	6.0 (4.0, 8.0)	7.0 (5.0, 8.0)	0.090
Motor	10.0 (7.0, 14.0)	12.0 (7.0, 15.0)	130 (11.0, 15.5)	0.166
Perseverence	6.0 (4.0, 8.0)	7.0 (5.0, 8.0)	7.0 (6.5, 8.5)	0.136
Self-control	8.0 (6.0, 13.0)	9.0 (6.0, 15.0)	13.0 (11.0, 17.0)	**0.034**
Cognitive complexity	11.0 (8.0, 14.0)	11.0 (7.0, 13.0)	11.0 (8.5, 12.0)	0.503
*Second order factors*				
Attentional	15.0 (9.0, 17.0)	15.0 (11.0, 19.0)	15.5 (13.0, 18.5)	0.146
Motor	16.0 (13.0, 21.0)	20.0 (14.0, 24.0)	20.5 (17.5, 23.5)	0.910
Non-planning	20.0 (14.0, 26.0)	22.0 (13.0, 29.0)	23.5 (21.0, 28.5)	0.144
**EuropASI Total**				
Medical	–	0.0 (0.0, 0.7)	0.0 (0.0, 0.7)	0.755
Job satisfaction	–	0.5 (0.0, 0.8)	0.5 (0.2, 1.0)	0.480
Alcohol use	–	0.0 (0.0, 0.0)	0.0 (0.0, 0.0)	0.710
Drug use	–	0.0 (0.0, 0.1)	0.3 (0.1, 0.4)	**0.001**
Legal status	–	0.2 (0.0, 0.4)	0.4 (0.3, 0.6)	**0.041**
Family	–	0.0 (0.0, 0.4)	0.0 (0.0, 0.2)	0.940
Social relations	–	0.0 (0.0, 0.4)	0.0 (0.0, 0.4)	1.000
Psychiatric status	–	0.0 (0.0, 0.4)	0.4 (0.1, 0.5)	0.093
**Heroin use (days of the previous month)**	–	–	27 (0, 30)	–
**Cannabis use (days of the previous month)**	–	–	20 (1, 30)	–
**Methadone dosage (mg)**	–	25.0 (18.0, 32.0)	28.0 (25.0, 33.0)	0.290
**s-COMT activity (pmol/mg prot/h)**	19.14 ± 4.12	17.98 ± 3.87	18.42 ± 4.19	0.707

Results are presented as median (Q1, Q3) or mean ± SD

*BIS-11* Barratt Impulsiveness scale version 11, *EuropASI* European version of the Addiction Severity Index, *s-COMT* soluble catechol-O-methyltransferase, *SUD* Substance use disorders

### Correlation between S-COMT erythrocyte activity and BIS-11

**Fig. 1:**
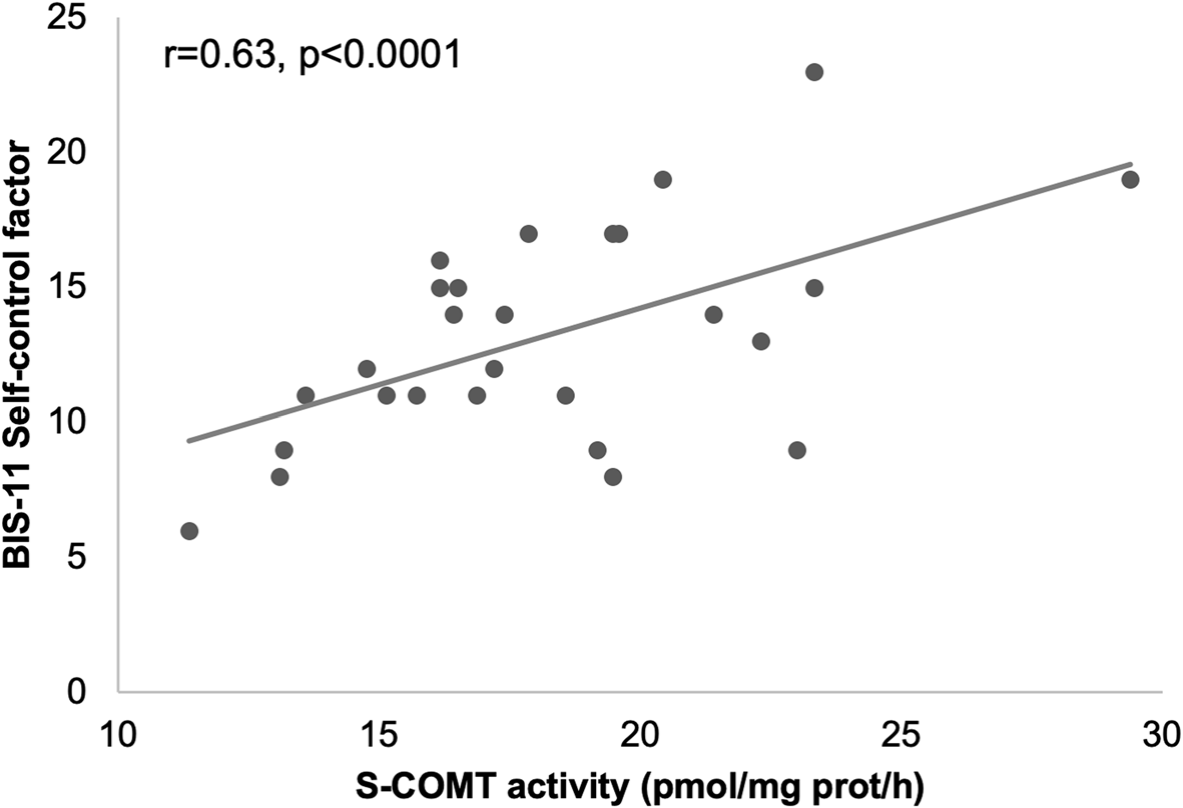
Correlation between S-COMT erythrocyte activity and the first order factor self-control (r = 0.63. *p* < 0.0001), in the SUD group

In the whole cohort and in SUD individuals (Fig. 1), s-COMT erythrocyte activity was correlated with the first order factor self-control (SUD r = 0.63, *p* < 0.0001; whole cohort =0.36, *p* = 0.015) and with the second order factor non-planning of BIS-11 (SUD r = 0.53, *p* = 0.002; whole cohort r = 0.29, *p* = 0.047). In the group of individuals without SUD no correlation between BIS-11 scores and s-COMT erythrocyte activity was observed (self-control r| = 0.014, *p* = 0.954; non-planning r = − 0.041, *p* = 0.866).

### Correlation between s-COMT erythrocyte activity and EuropASI

**Fig. 2:**
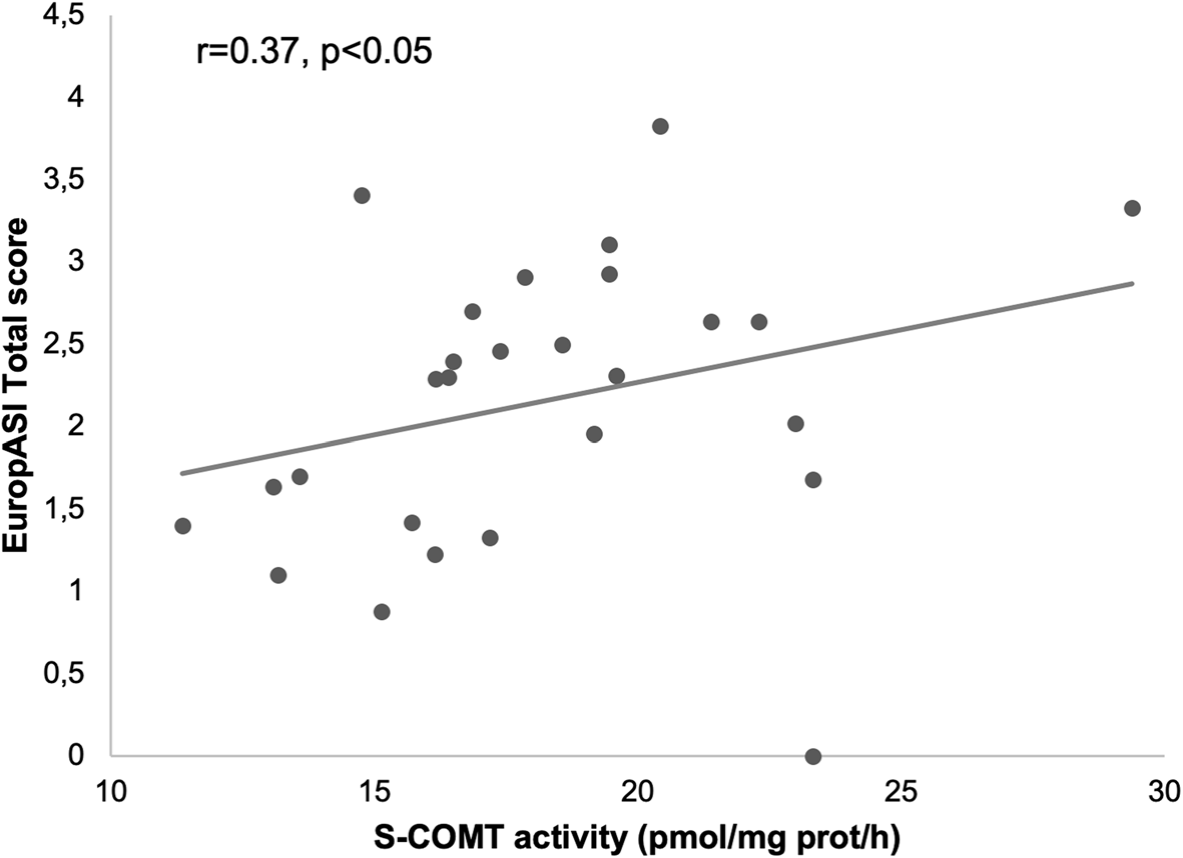
Correlation between the S-COMT erythrocyte activity the total score of EuropASI, in the SUD group

In the group of individuals with SUD, s-COMT erythrocyte activity was correlated with the total score of EuropASI (r = 0.38, *p* = 0.009) (Fig. 2).

## Discussion

This study is part of a broad project, which was implemented in a medium/high security penal institution for male offenders in the North of Portugal, with the objective of identifying aggression predictive factors among incarcerated individuals. On previous studies we evaluated the prevalence of premeditated and impulsive aggression in male offenders with ASPD (and determined the impact of impulsivity, SUD, psychopathy on aggression type) [35], and studied the association between s-COMT activity and premeditated aggression (submitted article).

In this study, we assessed s-COMT activity and impulsivity in male offenders with SUD and found that, in these individuals, s-COMT activity is correlated with self-control (BIS-11 first order factor) and non-planning (BIS-11 second order factor).

The profile of our overall population is similar to those described in recent reports of male prisons in developed countries: most individuals are below 40 years old, with low education level and moderate imprisonment length [20]. In terms of psychiatric disorders, most individuals were incarcerated due to violent crimes, presented ASPD and SUD, in close agreement with other evaluations performed in European prisons [36]. Anxiety disorders and suicide attempts rates were higher than those described before in similar populations [37]. We belief that these aspects can be related not only to specificities of the individuals, such as the mechanisms of dealing with the committed crime and with imprisonment, but also to the conditions of the prison in terms of facilities, medical and psychological care, and social dynamics. Regarding the prevalence of SUD, our results evidence the dimension of this problem in Portuguese prisons, with values up to 45% similar to those reported in other penal institutions worldwide [38].

The registered significant differences between the individuals with and without SUD regarding ASPD (Table 1, *p* = 0.038) are in good agreement with literature regarding the prevalence of this disorder among substance addicts [39]. This trend has been extensively explored in the last decades and research has shown that ASPD is one of the most common psychiatric diagnoses among individuals with SUD [40].

The evidenced significant difference between the non SUD and SUD participants (with or without active consumption) regarding factor 3 of the PCL-R illustrates the impact of this aspect of psychopathy on externalizing behaviors as showed before by Patrick and co-workers [41].

Our results showed also a significant difference, between the three groups, in the BIS first order factor self-control (Table 2, *p* = 0.034), which is not surprising considering the extensive evidence on this subject [42–44]. This trend is further reinforced by the calculated significant correlation between BIS-11 self-control factor and s-COMT activity in individuals with SUD (Fig. 1). In fact, COMT is a key factor in dopaminergic processes and, as such, this correlation is not surprising. Impulsive behaviours and self-control have been extensively studied through the measurement of s-COMT activity and the results evidence that enzyme levels correlate with levels of impulsivity [7, 9, 13]. This correlation has been confirmed by studies in which COMT inhibitors have proved to reduce impulsive addictive behaviors [45].

Our study showed also that s-COMT activity is correlated with the severity of the addiction measured by the EuropASI scale (Fig. 2). Even though this association has never been reported before, it confirms the dopaminergic pathways of substance addiction [6, 13]. However, we could not find differences in s-COMT activity or COMT polymorphisms in SUD and non-SUD patients (Table 2). In addition, active substance consumption did not show to affect COMT activity nor the severity of dependence (Table 2). These results seem to direct the attention to impulsivity as major determinant for SUD, as evidenced by the already mentioned differences in the BIS-11 self-control and non-planning factors (Table 2).

This study presents some limitations. First, this is a monocentric study including participants from only one penal institution. This kind of study tends to carry a higher probability of not finding differences between groups and can have type 2 errors. Second, our sample is small and thus our results shall be confirmed in a more expressive number of subjects. However, sample size shall be evaluated considering the particularities of this specific population and the limitations regarding the contact with participants, consultations, and research approval. These facts alerted us for the need to create adequate conditions to perform research inside prisons.

In conclusion, our study evidenced, for the first time, a correlation between the severity of substance dependence and s-COMT activity and highlighted the correlation between non-self-control and COMT activity, in individuals with SUD. This further opens new perspectives regarding the pharmacological intervention on SUD through the interference on the dopamine pathways catalysed by COMT. The replication of the study in non-prison settings could further extend the findings to the general population.

## Data Availability

Original datasets are available in a publicly accessible repository: The original contributions presented in the study are publicly available. This data can be found here: https://datadryad.org/stash/share/YNn2Rt_VawfkndMH3NPkYc-YLytKZ3zDrF33S345RHg

## Abbreviations

ASPD: Antisocial personality disorder
BIS-11: Barratt Impulsiveness scale version 11
COMT: Catechol-O-methyltransferase
EuropASI: European version of the Addiction Severity Index
MINI: Mini-International Neuropsychiatric Interview
PCL-R: Psychopathy Checklist-Revised
PFC: Prefrontal cortex
SUD: substance use disorders
